# Assessment of Cosmetic and Dermatological Properties and Safety of Use of Model Skin Tonics with Kombucha-Fermented Red Berry Extracts

**DOI:** 10.3390/ijms232314675

**Published:** 2022-11-24

**Authors:** Aleksandra Ziemlewska, Zofia Nizioł-Łukaszewska, Martyna Zagórska-Dziok, Magdalena Wójciak, Dariusz Szczepanek, Ireneusz Sowa

**Affiliations:** 1 Department of Technology of Cosmetic and Pharmaceutical Products, Medical College, University of Information Technology and Management in Rzeszow, Sucharskiego 2, 35-225 Rzeszow, Poland; 2 Department of Analytical Chemistry, Medical University of Lublin, Aleje Raclawickie 1, 20-059 Lublin, Poland; 3 Chair and Department of Neurosurgery and Paediatric Neurosurgery, Medical University of Lublin, 20-090 Lublin, Poland

**Keywords:** *R. rubrum* L., *F. vesca* L., *R. idaeus* L., kombucha, polyphenols, skin cells, antioxidants, skin hydration, skin tonic

## Abstract

Kombucha is a health-promoting beverage that is produced by fermenting sweetened tea using symbiotic cultures of bacteria belonging to the genus Acetobacter, Gluconobacter, and yeast of the genus Saccharomyces. This study compared the cosmetic and dermatological properties of the extracts of the following redberries: *R. rubrum, F. vesca,* and *R. idaeus,* and their ferments, which were obtained by fermentation for 10 and 20 days using tea fungus. For this purpose, the fermented and non-fermented extracts were compared in terms of their chemical composition using the HPLC/ESI-MS chromatographic method, demonstrating the high content of biologically active compounds that were present in the ferments. The antioxidant activity of the tested samples was evaluated using DPPH and ABTS tests, as well as by evaluating the scavenging of the external and intracellular free radicals. The cytotoxicity of the extracts and the ferments, as well as the cosmetic formulations, were also determined by conducting Alamar Blue and Neutral Red tests assessing the cell viability and metabolism using skin cell lines: fibroblasts and keratinocytes. In addition, application tests were conducted showing the positive effects of the model cosmetic tonics on the TEWL, the skin hydration, and the skin pH. The results indicate that both the extracts and the ferments that were obtained from kombucha can be valuable ingredients in cosmetic products.

## 1. Introduction

Year after year, the cosmetics industry seeks new solutions and raw materials in order to keep up with user expectations and to meet the demands of increasingly conscious customers. For years, a clearly noticeable trend persisting in the production of cosmetics are products that are of natural origin. The raw materials that were used years ago are returning to favor, but in a completely different formula. Scientists around the world are looking for new technologies for the use of raw materials in the name of the “zero waste” principle [1]. One alternative that fits into this principle is fermentation. This process has been known for a long time, and it leads to the enrichment of raw materials with biologically active compounds, which are gaining more and more importance in the cosmetics industry every year. The cosmetics that are created using fermentation are referred to as bioferments. Thanks to the use of microorganisms in this process, the resulting products are characterized by increased bioavailability and contain a number of beneficial compounds, such as antioxidants, ceramides, proteins, and amino acids [2].

Fermentation with symbiotic cultures of bacteria and yeast (SCOBY) is particularly noteworthy, which was made known through kombucha, but is now gaining wider application in the fermentation of a variety of biosources [3]. Kombucha owes its unique properties to bioactive compounds, including the vitamins, the minerals, and the amino acids that are contained in black or green tea, as well as a number of active compounds, such as polyphenols, that are formed by their hydrolysis during the fermentation process [4]. It is extremely important that the properties of the resulting ferments depend on the consortium of microorganisms, but also on the plant material that is being fermented. Consequently, the right choice of fermented substrates can help us to achieve the sought-after properties or characteristics of the product. The cosmetics industry is increasingly turning to natural plant substrates that were previously considered to be waste products, such as leaves or fruit seeds. Subjecting them to a fermentation process has yielded surprising discoveries of properties that are quite different from those known in fruits [5]. The same strategy is also used in the production of biosurfactants, which are characterized by higher activity and stability compared to the surfactants that are obtained synthetically. Potato peelings or sunflower hulls are used as substrates for this purpose, which significantly minimizes the production costs [6].

The properties of the fruit are well known and are widely used in both the pharmaceutical and the cosmetic industries. Berries showing anti-cancer and anti-viral effects, as well as those providing relief from atopic dermatitis, have been widely studied [7,8]. However, subjecting berries to a fermentation process can result in different or enhanced effects, which is why it is important to seek innovative methods of extracting them [9]. Studies show that the color of fruits can define their properties. Anthocyanins, which are considered to be among the most potent antioxidants, reducing the number of free radicals, the accumulation of which contributes to faster aging, are responsible for the dark color of fruits. The high content of these pigments also has an antibacterial and anti-inflammatory effect, and strengthens the blood vessels [10]. All of the fruits that have been analyzed in this work (*R. rubrum, F. vesca,* and *R. idaeus*) are characterized by a high content of anthocyanins, which show strong antioxidant activity and high levels of vitamin C [11,12]. *Ribes rubrum* shows anti-inflammatory, anti-diabetic, and antioxidant effects [13]. *Fragaria vesca* is known for its anti-cancer, anti-inflammatory, and anticoagulant properties [14]. *Rubus idaeus*, on the other hand, has shown anti-inflammatory and protective effects against UVB radiation [15].

These properties are used in cosmetics to slow down skin aging, to protect against UV radiation, and to seal the blood vessels [16]. Thus, the use of cosmetics containing in their composition the extracts of fruits that are rich in these pigments levels skin irritation and redness, reduces the appearance of hyperpigmentation and dark circles, and has moisturizing, soothing, brightening, and rejuvenating effects [17]. Carotenoids, which are known for their ability to absorb UV radiation, and thus have a protective function for the skin, also exhibit antioxidant effects [18]. Research into increasing the production of pigments in fruits, and thus increasing their health-promoting qualities, is becoming increasingly common [19]. Thanks to the presence of retinoids and polyphenols in fruits, cosmetics that are based on these raw materials show moisturizing, smoothing, and soothing effects [20]. Polyphenols have an important function in inhibiting collagenase and elastase activity, thereby preventing collagen hydrolysis [21]. Moreover, they reduce skin irritation and redness, have a positive effect on skin microcirculation, and protect against adverse external factors, including UV radiation [22]. Retinoids, on the other hand, reduce transepidermal water loss (TEWL) by strengthening the epidermal barrier, which has a positive effect on skin hydration [23].

Fruit extracts and ferments can have a beneficial effect on the skin microbiome and regulate the processes occurring in it. Studies confirm that fruits exhibit anti-inflammatory and antioxidant effects, among others, indicating their important role in conditions such as atopic dermatitis, skin sores, acne vulgaris, rosacea, photoaging, and psoriasis [24]. More and more researchers are also focusing on the link between the skin microbiome and the gut, which is referred to as the gut–skin axis (GSA), and its impact on dermatological conditions [25]. It has also been shown that certain skin lesions can correlate with the severity of intestinal inflammation [26]. The versatility of the fruit’s use allows it to be used both in cosmetics that are applied directly to the skin, as well as in dietary supplements, acting “from inside”.

In order to investigate the properties of the extracts and kombucha ferments, the content of the biologically active compounds was determined, and the antioxidant properties were examined. Furthermore, the extracts and ferments were tested for cytotoxic activity on fibroblasts and keratinocytes. The obtained extracts and ferments were used as the active ingredient of model skin tonics, in order to increase their safety of use. For the obtained cosmetics containing the analyzed extracts and ferments, and the reference sample (without the addition of the extracts and the ferments), the effect on skin hydration and the pH, as well as transepidermal water loss from the epidermis (TEWL), were determined.

## 2. Results and Discussion

### 2.1. Determination of Bioactive Compounds

**Figure 1:**
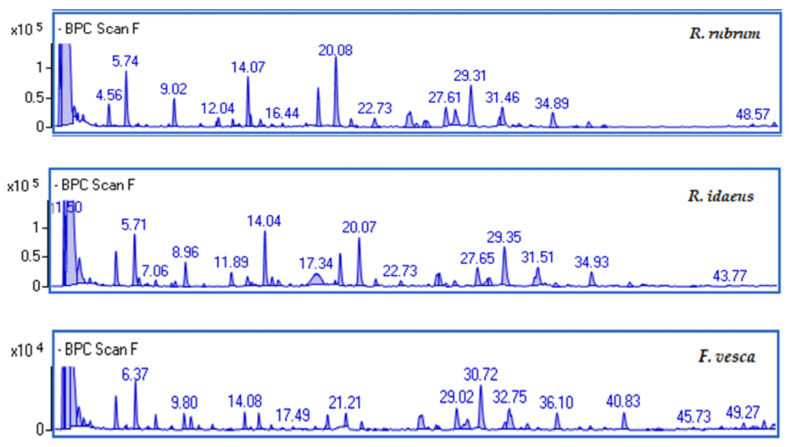
Representative base peak chromatograms (BPC) for *R. rubrum, R. idaeus,* and *F. vesca*.

UHPLC DAD/TOF MS was used for spectral and chromatographic analysis and quantification. The main compounds were characterized using electrospray ionization mass spectrometry (TOF) in the positive ionization mode for the anthocyanins and in the negative ionization mode for the flavonols, the phenolic acids, and flavon-3-ol. The mass data were confirmed by the comparison mass that was given in the literature or with the standards. The berry fruit chromatograms are presented in Figure 1. The summary of all of the identified phenolic compounds for each sample type, i.e., *R. rubrum, R. idaeus*, and *F. vesca*, is presented in Table 1. The details of MS identification are presented in Tables S1–S3 in the Supplementary Material. The profile that was obtained was similar to those reported in the literature. As demonstrated in previous scientific studies, the fruits of *R. rubrum, R. idaeus,* and *F. vesca* are rich in phenolic compounds with health-promoting properties [27]. Moreover, kombucha-fermented berry extracts are distinguished by their significant content of biologically active compounds. Table 2 compares the contents of the compounds in terms of their chemical composition. The obtained results are expressed in µg/mL of the extract. The research indicates that, during the fermentation process of Kombucha, there is an increase in the content of certain phenolic compounds, and that the fermentation time influences the growth and the content of these compounds. The labelled polyphenolic compounds are mostly presented in F10 and F20, while the extracts do not have them at all. This may indicate that the berry extracts are less stable in terms of their polyphenol content than their kombucha-ferments. The differences between the fermented and the non-fermented extracts may also be due to the fact that complex phenolic compounds can be degraded to smaller molecules during fermentation [28,29]. It was found that, for *R. idaeus* fruit, the most abundant compound was salicylic acid glucoside (10.95 µg/mL ± 0.06 for F20). In contrast, *R. rubrum* is characterized by a high content of benzoic acid hexoside (3.92 ± 0.20 µg/mL for the extract and 3.78 µg/mL ± 0.12 for F10). Furthermore, differences in the polyphenol content were observed between the extracts and their ferments. For example, for *F. vesca*, the gallic acid content of F20 is about 12 times higher than that of the extract. An earlier paper published by Ziemlewska et al. [5] determined the content of the biologically active compounds that are present in berry leaf extracts, which are often considered to be agro-waste. Compared to berry fruits, leaf extracts and their kombucha ferments at the same tested concentration are characterized by a higher content of polyphenols, which also translates to the antioxidant activity. These differences are significant in terms of the health-promoting properties and their potential use.

### 2.2. Assessment of Antioxidant Activity

Tackling the risks that are caused by reactive oxygen forms can take place on many levels; therefore, it should be considered that the human body is constantly exposed to adverse external factors. One of the effects of this type of action may be the excessive production of free radicals, the effect of which is, among others, premature skin aging. An appropriate line of defense is the use of antioxidants, thanks to which it is possible to reduce the impact of adverse free radical reactions on the body. It should be noted that many products of plant origin exhibit strong antioxidant properties with the ability to neutralize ROS, i.e., reactive oxygen species [30]. These raw materials include berries that contain antioxidants, in particular polyphenolic compounds, which are the source of antioxidant compounds, including anthocyanins, ascorbic acid, phenolic acids, and flavonoids [31,32]. Research confirms that the cultivars of the tested berries show significant antioxidant activity, e.g., by scavenging hydroxyl radicals, singlet oxygen, hydrogen peroxide, and superoxide radicals. Flavonoids (mainly quercetin and rutin) have been shown to protect vitamin C and E, and their ability to chelate copper ions and other transition metals inhibits ascorbate oxidation. In addition, substances with antioxidant activity may have a beneficial effect on the condition of the skin, and also play a role of auxiliary substances that affect the durability or the bioavailability of the preparation [33,34,35].

During this study, the antioxidant potential of the analyzed *R. rubrum*, *R. idaeus*, and *F. vesca* extracts, and the resulting ferments that were obtained after 10 and 20 days, was assessed. In order to evaluate the antioxidant properties, two different assays were used in order to avoid possible irregularities in the performance of the tested extracts, given the differences in their principles of action. The antioxidant potential of all of the tested samples was evaluated by DPPH and ABTS assays. The measure of the antioxidant activity is the value of the IC_50_ parameter, which determines the concentration of the plant extract and the kombucha ferments that causes a 50% decrease in the initial radical concentration. The antioxidant properties of the extracts and the ferments were tested over a concentration range of 30 µg/mL to 300 µg/mL. The IC_50_ results for the DPPH and ABTS assays are shown in Table 3 and Table 4.

Table 3 presents the values of IC_50_ of the DPPH radical scavenging for three types of analyzed plants for their extract, as well as 10- and 20-day ferments. The first observation is that the ability to inhibit the DPPH radical is statistically higher for the ferments compared to the extracts. Among the pure non-fermented extracts, the lowest value was observed for *F. vesca*. When attention was focused on the extracts, then the most preferable and lowest values for both 10- and 20-day ferments were observed for *R. rubrum*, being 5 to almost 9% lower than the values for *F. vesca* and *R. idaeus* in the case of the 10-day ferment, and 3 to 6% lower in the case of the 20-day ferment. The lowest value among all of the investigated cases was obtained for *R. rubrum* for the 10-day ferment. Table 4, again, presents the values of IC_50_, but this time it depicts the ABTS+ radical scavenging for the three types of analyzed plants for their extract, as well as the 10- and 20-day ferments. It is shown that the ability to inhibit the ABTS radical is statistically higher for the ferments compared to the extracts. What can be noticed is that the value that was obtained for extract of *R. rubrum* gave a significantly higher value compared to the two other plants. It is worth mentioning that the situation changes when we analyze the ferments, where *R. rubrum* becomes a preferable plant in the case of the 10-day ferment, with values about 1 to 3% lower than that of the two another plants. In the case of the 20-day ferments, the most preferable values were observed for *R. idaeus*, being 3 to 5% lower than the others, which is the lowest value among all of the investigated cases.

**Figure 2:**
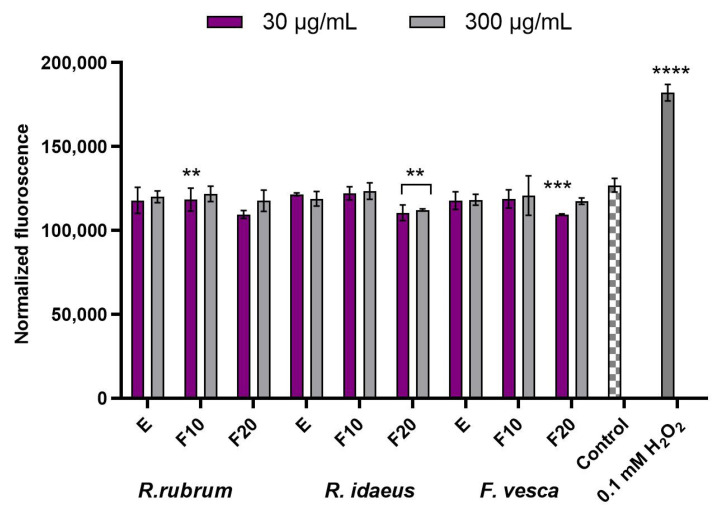
Effect of *R. rubrum, R. idaeus*, and *F. vesca* extracts and kombucha ferments (at concentrations of 30 µg/mL and 300 µg/mL) on the DCF fluorescence in fibroblasts (BJ). Data are the mean ± SD of three independent experiments. **** *p* < 0.0001, *** *p* < 0.001, ** *p* < 0.01 compared to the control.

**Figure 3:**
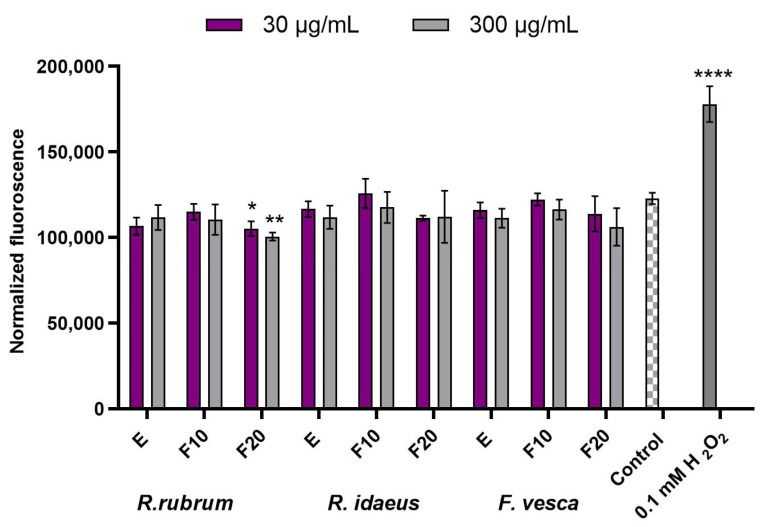
Effect of *R. rubrum, R. idaeus*, and *F. vesca* extracts and kombucha ferments (at concentrations of 30 µg/mL and 300 µg/mL) on the DCF fluorescence in keratinocytes (HaCaT). Data are the mean ± SD of three independent experiments.**** *p* < 0.0001, ** *p* < 0.01, * *p* < 0.05 compared to control.

In the next stage of the research, the ability of the extracts and the ferments to reduce the intracellular production of reactive oxygen species (ROS) on keratinocyte (HaCaT) and fibroblast (BJ) cell lines was assessed. The analysis showed that both fibroblasts and keratinocytes had the potential to reduce the intracellular ROS production by the extracts and ferments at both of the tested concentrations (30 µg/mL and 300 µg/mL). In Figure 2, the effect of the analyzed plant extracts and their ferments on the DCF fluorescence in fibroblasts has been investigated. First, one can notice that all of the measured values are noticeably lower when compared to the control. In addition, in a huge majority of the examined combination of plant and extract or ferment, the observed values of the normalized fluorescence were higher in the case of the higher concentration of 300 µg/mL when compared to the lower concentration of 30 µg/mL. There was only one single exception from that observation for the extract from *R. idaeus*, where a slightly lower value for the higher concentration was observed. Another observation is that, when analyzing a given plant for a given concentration, each of the case values observed for the 10-day ferments were higher than the values that were observed for the extract; however, for the 20-dayferment, the values dropped below the values that were observed for the extracts. When we focus on looking for the lowest values, then all three of the examined plants for the 20-day ferments in the lower concentration had similar results, with a slightly lower value for *F. vesca*. It looks slightly different when we focus on the 20-day ferments in the higher concentration, then the lowest value is observed for *R. idaeus*, but the value is still slightly higher than that in the lower concentration. In Figure 3, a similar experiment has been summarized, where keratinocytes have been used instead of fibroblast. In this case, most of the results are smaller when compared to the control, with the only exception for the *R. idaeus* 10-day ferment in a concentration of 30 µg/mL (higher value) and *F. vesca,* also in case of the 10-day ferment in concentration of 30 µg/mL (the same value). Next in this experiment, the values that were observed for the higher concentration were smaller compared to the values observed for the lower concentration, with only one exception for the extract of *R. rubrum*, where the opposite effect was observed. When comparing the plants between themselves, *R. rubrum* showed the lowest values when compared with other plants within the same combination of extract or ferment and concentration. In addition, when we focus on looking for the lowest values, then *R. rubrum* looks the most promising, showing the lowest observed values for the 20-day extract in the higher concentration. When comparing *R. idaeus* and *F. vesca* for the case of the extracts, the values that were observed for the extracts were very close, but in case of the 10-day ferment, the values for *R. idaeus* were higher.

It is worth mentioning that, in the discussed experiment, the antioxidant potential was growing with time for all three of the analyzed plants. Other authors also report an increase in the antioxidant potential with increasing fermentation time, which was also observed in the case of tea fermentation [34]. The antioxidant activity of kombucha has been described as being mainly due to the catechins and the polyphenols that are present in the tea (*C. sinensis*) leaves. The reason for the higher antioxidant activity of kombucha tea compared to non-fermented tea is that the polyphenolic substances in the tea are broken down into smaller phenolic substances by microbial enzymes in the biofilm that is formed by bacteria and yeast during the fermentation, therefore, the total amount of phenols increases. Furthermore, one reason for the increase in the total flavonoids after fermentation is that these enzymes can reduce the polyphenolic substances to flavonoids. It has also been shown that, in addition to the polyphenols, the kombucha-fermented plant extracts are rich in vitamins (B1, B2, B6, B12, and C) and organic acids (i.e., acetic, gluconic, glucuronic, lactic, tartaric, citric, and malic acids), which contribute to the antioxidant activity [36,37].

### 2.3. Cytotoxicity Assessment

The assessment of the influence of the extracts and the ferments with potential cosmetic use on the viability, the metabolic activity, and the integrity of skin cell membranes is an extremely important aspect in the assessment of their cytotoxicity. As part of the work, two tests that are commonly used in the assessment of the cytotoxicity of plant materials were used. The first of them, which is the Alamar Blue (AB) assay, allows the assessment of the viability of the tested cells by assessing the functioning of the mitochondrial respiratory chain [38]. The second (Neutral Red assay) is based on the detection of viable cells by assessing the uptake of the Neutral Red dye, which stains the lysosomes in living cells. The amount of dye that is released from the cells under the acidification and extraction conditions is used to determine the total number of viable cells and, thus, the cytotoxicity of the test compound [39].

**Figure 4:**
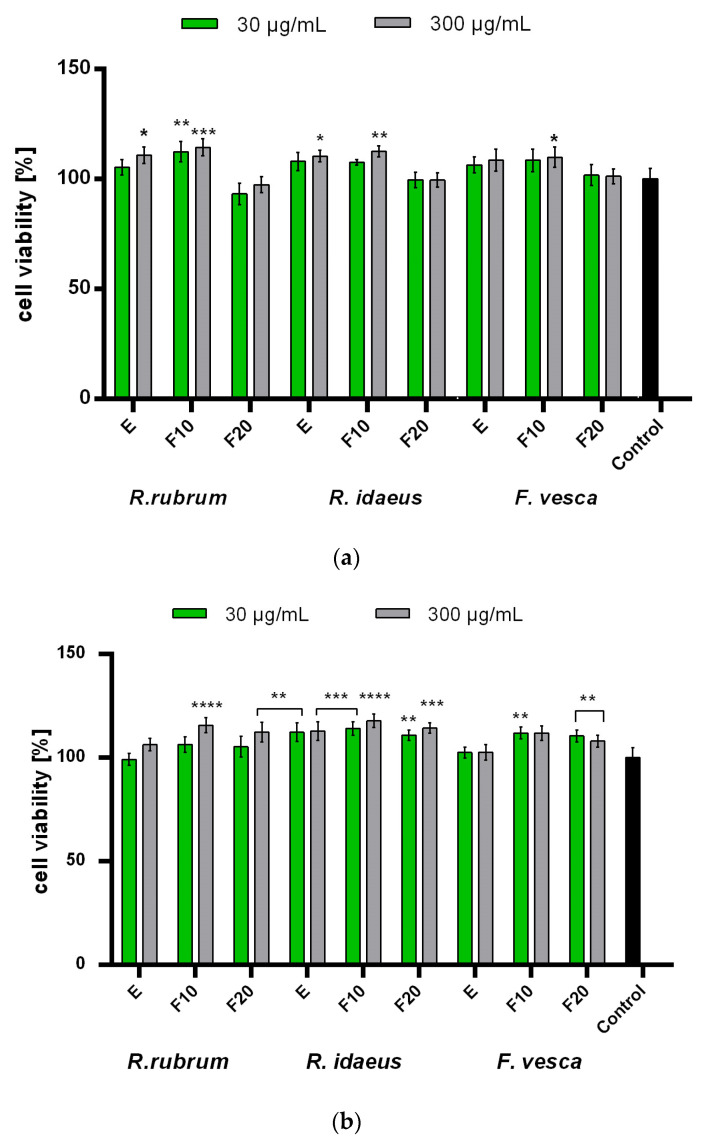
Effect of *R. rubrum, R. idaeus,* and *F. vesca* extracts and ferments (at the concentration of 30 and 300 μg/mL) on Alamar Blue Assay (**a**) and Neutral Red dye uptake (**b**) in cultured fibroblasts (BJ) after 24 h of exposure. Data are the mean ± SD of three independent experiments, each consisting of three replicates per test group. **** *p* < 0.0001, *** *p* < 0.001, ** *p* < 0.01, * *p* < 0.05 compared to control.

In order to assess the effect of the tested extracts and the ferments on both epidermal and dermal cells, analyzes were performed on keratinocytes (HaCaT) and fibroblasts (BJ). The conducted analyzes showed no statistically significant cytotoxicity of all of the tested extracts and ferments on skin cells in vitro. The results that were observed in the case of applying the extracts and the ferments from *R. rubrum*, *R. idaeus*, and *F. vesca* to the cells were similar and no major differences were observed between the plants that were tested. In the case of the fibroblasts that were subjected to the Alamar Blue test, it was shown that the extracts and the ferments after 10 days of fermentation increase the viability and the metabolic activity of these cells. It was also shown that this effect depends on the concentration of the tested extracts and ferments. It was also observed that the ferments that were obtained after a longer fermentation time (20 days) slightly inhibited the viability of these cells. In studies that aimed to assess the possibility of NR dye accumulation in fibroblast lysosomes, it was shown that both the extracts and the ferments that were obtained after both fermentation times show a positive effect on the viability of these cells (Figure 4).

**Figure 5:**
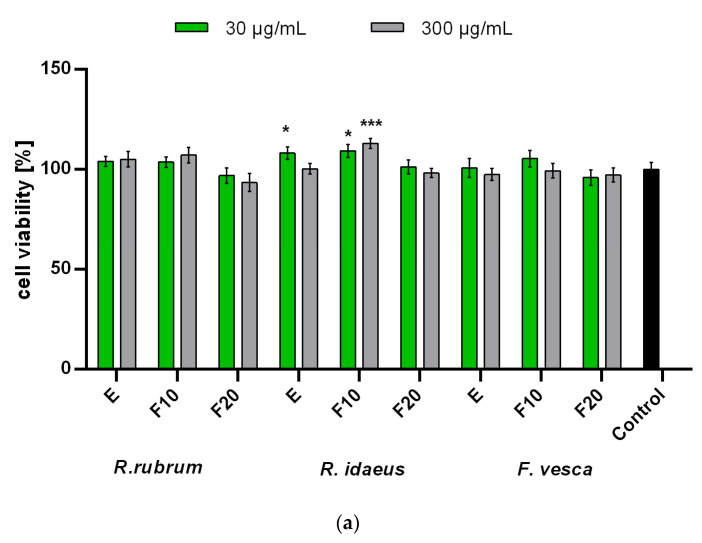

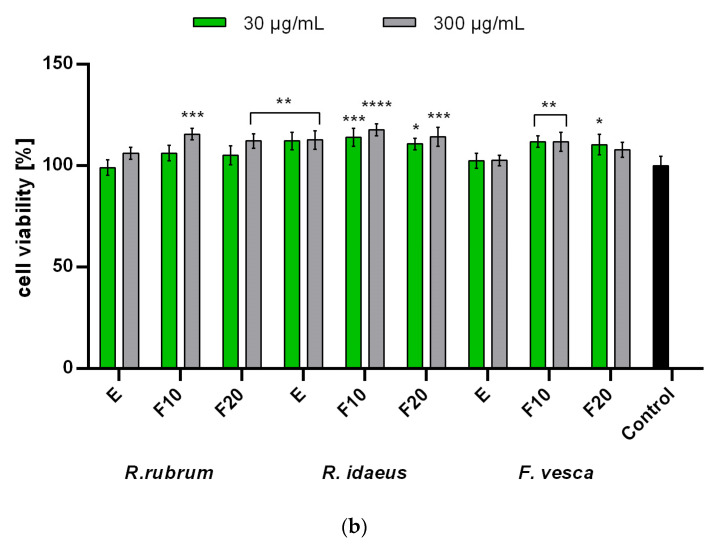
Effect of *R. rubrum, R. idaeus,* and *F. vesca* extracts and ferments (at the concentration of 30 and 300 μg/mL) on Alamar Blue Assay (**a**) and Neutral Red dye uptake (**b**) in cultured keratinocytes (HaCaT) after 24 h of exposure. Data are the mean ± SD of three independent experiments, each consisting of three replicates per test group. **** *p* < 0.0001, *** *p* < 0.001, ** *p* < 0.01, * *p* < 0.05 compared to control.

**Figure 6:**
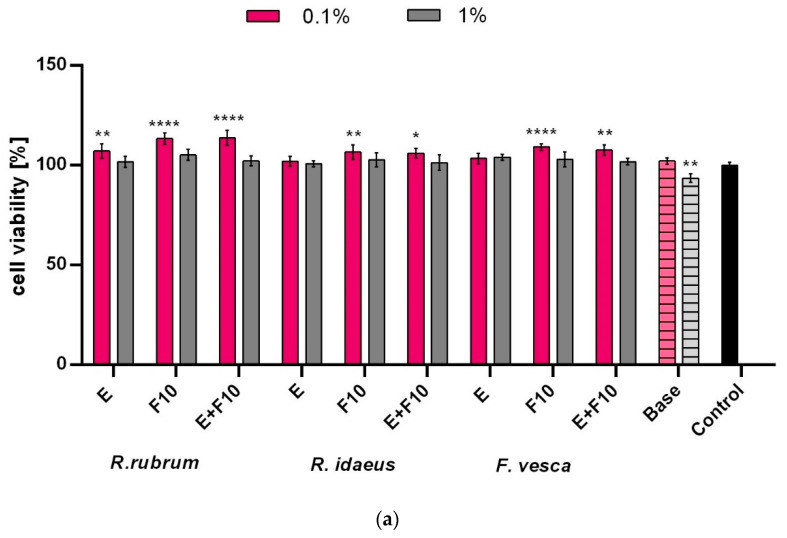

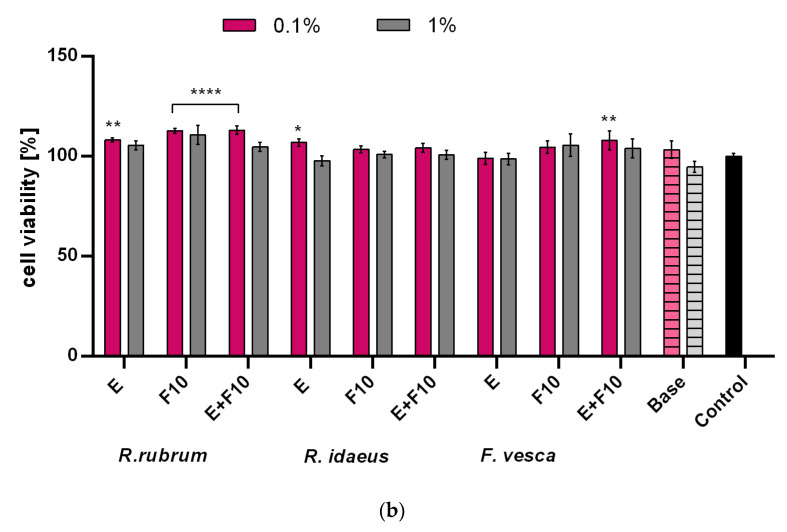
Effect of cosmetic formulation containing *R. rubrum, R. idaeus,* and *F. vesca* extracts and ferments (at the dilution of 0.1 and 1%) on Alamar Blue Assay (**a**) and Neutral Red dye uptake (**b**) in cultured keratinocytes (HaCaT) after 4 h of exposure. Data are the mean ± SD of three independent experiments, each consisting of three replicates per test group. **** *p* < 0.0001, ** *p* < 0.01, * *p* < 0.05 compared to control.

In the case of the keratinocytes that were subjected to the AB test, no significant decrease in the viability of the tested cells was observed, the viability of which, after using both the extracts and the ferments, was similar to that of control cells. In the case of these cells, only the extract and the F10 ferment from *R. idaeus* statistically significantly increased the viability of these cells, while in the case of the other plants, this effect was not observed. On the other hand, the NR test showed that the ferments (both F10 and F20) of all of the tested plants have a positive effect on the viability and the integrity of keratinocyte cell membranes, enabling the accumulation of the dye that is used in lysosomes. This effect was not observed for the extracts of *R. rubrum* and *F. vesca*, which indicates a more favorable effect of the ferments than the extracts of these plants (Figure 5). It is also worth noting that the addition of the tested extracts, ferments, and their combinations to the obtained cosmetic formulations reduces the cytotoxicity of these cosmetics. As shown in Figure 6, the viability of the keratinocytes that were treated with the designed formulations was most favorably influenced by *R. rubrum* extracts and ferments, which are able to increase the viability of the keratinocytes by almost 20%. These results show that the addition of the extracts and the ferments from all of the tested plants, in addition to enriching the cosmetic preparations with many biologically active compounds, also has a positive effect on skin cells.

The positive effect of the tested ferments and extracts on the metabolism and the viability of skin cells is certainly the effect of many biologically active compounds that occur in the extracts and ferments that were obtained in this study. The compounds whose presence has been confirmed in chromatographic analyzes have been proven to have protective properties against skin cells. Chen et al. pointed out that chlorogenic acid has strong antioxidant properties through the possibility of increasing the activity of superoxide dismutase, catalase, and glutathione. This compound can also reduce lipid peroxidation, which also plays an important role in the protection of cells [40]. Other authors also report that this compound can be used in order to restore the impaired dermal matrix network, as well as the epidermal barrier [41]. Yang et al. demonstrated that gallic acid can protect skin cells by increasing the expression of antioxidant genes, accelerating the migration of the keratinocytes and the fibroblasts, as well as influencing the wound-healing processes by activating the focal-adhesion kinases (FAK) and c-Jun N-terminal kinases (JNK), as well as the extracellular signal regulated kinases (Erk) [42].

Thus, a more positive effect of the ferments that were obtained from the three tested plants compared to the extracts may result from the greater amount of biologically active compounds. An example of this is the catechins and their derivatives, the presence of which was found only in the obtained ferments. The beneficial effect of catechins on skin cells has been proven in numerous studies, which indicate that these compounds reduce skin damage, have a strong antioxidant effect, and may delay the degradation of the extracellular matrix that is caused by both ultraviolet radiation and pollution [43,44]. These compounds can also activate collagen synthesis and inhibit the production of the matrix metalloproteinase enzymes, which may contribute to slowing down the skin aging processes [45]. Tanigawa et al. also indicated that the treatment of fibroblasts with catechin may inhibit apoptosis resulting from the action of hydrogen peroxide and reduce JNK and p38 phosphorylation and the activation of caspase-3 [46]. The cytoprotective effect of the ferments on fibroblasts and keratinocytes, which has been proven in this work, may also be the effect of rutin, which has strong antioxidant properties and may prevent the increase in phosphatidylethanolamine and phosphatidylcholine levels in skin cells that are exposed to UVA/B radiation [47]. Gęgotek et al. show that this compound may also counteract the increase in phospholipase A2 activity, the intracellular production of ROS, and lowering the level of arachidonic and linoleic acid. Rutoside can also effectively prevent the decrease in the activity of glutathione peroxidase and glutathione, as well as protect against an increase in the level of the lipid peroxidation product [48]. Thus, the fermentation of raw materials, such as *R. rubrum, R. idaeus* and *F. vesca,* using a symbiotic culture of bacteria and yeast, known as kombucha, may contribute to obtaining ferments that are rich in a wide range of compounds, which exhibit extremely valuable properties, including protective effects and increased proliferation and metabolic activity of skin cells.

### 2.4. Transepidermal Water Loss (TEWL), Skin Hydration, and Skin pH Measurements

Plant extracts are considered to be a rich source of biologically active compounds with anti-aging antioxidant or anti-inflammatory properties. Increasingly, they are becoming the components of cosmetic preparations that are applied to the skin, thus contributing to an increase in skin hydration and preventing excessive water loss from the epidermis [49]. Proper skin hydration plays an important role in many processes of wound healing and skin regeneration, as well as in delaying skin aging [50]. In addition, recently, fermented plant extracts have gained importance as a source of active substances with a broad spectrum of action. Due to their content of simple chemical compounds of low molecular weight, plant ferments are a source of bioavailable active substances, which have a high rate of penetration into the deeper layers of the skin [51].

**Figure 7:**
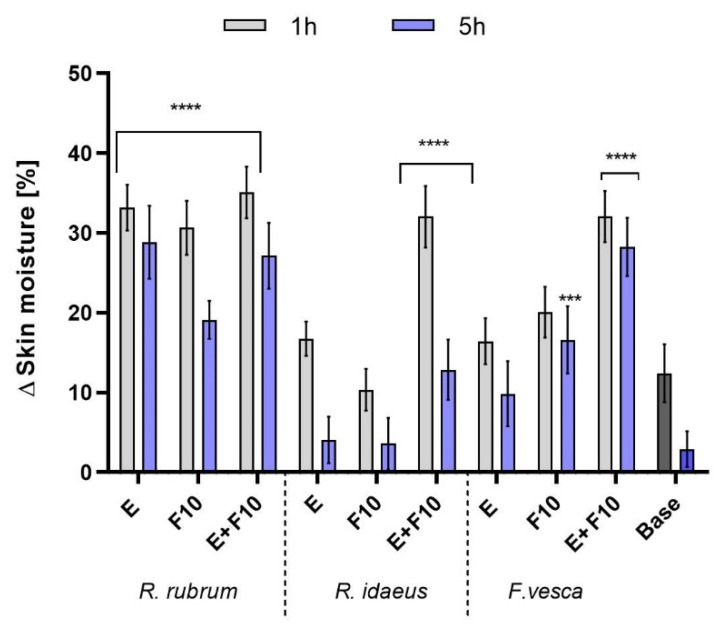
The influence of model skin tonics with *R. rubrum, R. idaeus,* and *F. vesca* extracts (E), ferments (F10), and extracts + ferments (E + F10) on skin hydration. Data are the mean ± SD of three independent measurements. **** *p* < 0.0001, *** *p* < 0.001 compared to the base tonic.

**Figure 8:**
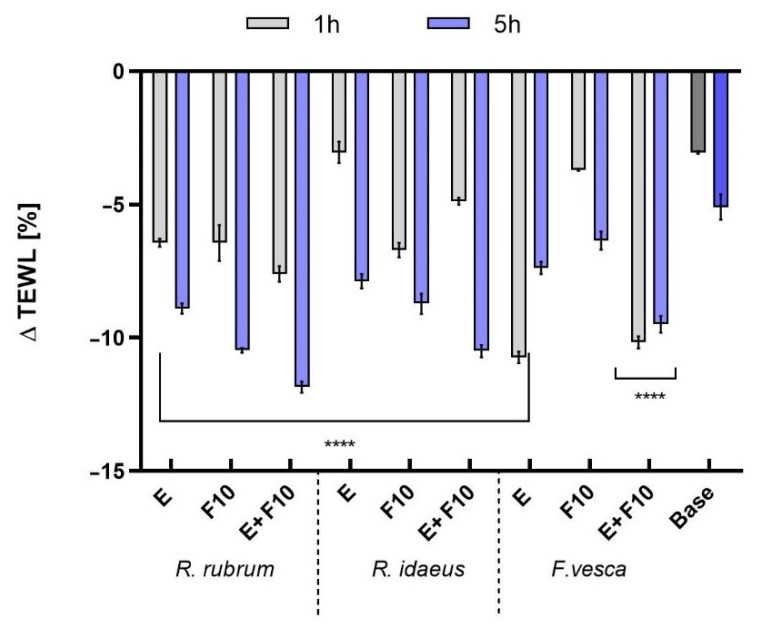
The influence of model skin tonics with *R. rubrum, R. idaeus,* and *F. vesca* extracts (E), ferments (F10), and extracts + ferments (E + F10) on transepidermal water loss (TEWL). Data are the mean ± SD of three independent measurements. **** *p* < 0.0001 compared to the base tonic.

In this study, hydration and transepidermal water loss (TEWL) analyzes were carried out, assessing the effects of the tested model skin tonics with extracts and kombucha ferments on the skin. The measurements were taken at two time intervals of one hour and five hours. The results are presented in Figure 7 and Figure 8. The model cosmetic tonics contained the tested plant extracts, kombucha ferments, and a mixture of extract and ferment after 10 days of fermentation. F10 was selected because these test ferments had a more favorable effect on skin cell proliferation than F20. The products that were tested contained 1% extract/ferment (*R. rubrum, R. idaeus, F. vesca)*. The model tonic that was designated as a base did not contain the tested extracts and ferments. The results are expressed as a percentage of the control field (without any tested skin tonic). This study showed that all of the model cosmetic tonics showed a skin moisturizing effect compared to the control field (without any tonic being tested). For all of the tested plants, the formulations containing extract and/or ferment showed a more favorable moisturizing effect compared to the base tonic. A correlation of decrease in skin hydration with time was observed for all of the tested tonics. For all of the plants that were tested, the combination of extract and ferment gave the most favorable skin moisturizing effect. Moreover, *R. rubrum* showed the best performance, obtaining almost three times higher moisturizing effect compared to the base tonic (for E + F10 after one hour of application). It was also noted that the application of the tested plant extracts and ferments had a positive effect on the TEWL level. The formulations with extract and kombucha ferment showed a decrease in the TEWL levels (compared to the control field). Moreover, by prolonging the exposure time of the tonics on the skin, differences were noted in preventing transepidermal water escape from the epidermis. The most favorable values in the level of TEWL were observed in the case of the tonic containing a combination of *R. rubrum* extract and ferment (about a 2.5 higher level of prevention of transepidermal water escape from the epidermis compared to the base tonic after five hours of the application). This study showed that preparations containing kombucha ferments had a more favorable effect on skin hydration compared to the skin tonics containing 1% of plant extracts. The simple sugars that accumulated during the fermentation process contain hydroxyl groups in their structure and are, therefore, valuable humectants that have a greater ability to penetrate deep into the epidermis than complex substances, which act mainly on the skin surface [52]. The HPLC analysis of redberry extracts and kombucha ferments, which were described earlier, revealed a rich content of phenolic compounds and flavonoids, which, in addition to being known antioxidants, have beneficial effects on the skin [53]. Other studies have also shown the effectiveness of red berries in promoting skin hydration and protecting cells from excessive water loss. Thanks to these actions, the plant extracts have good potential for use in skin care products, especially those that are specifically intended for dry and aging skin [54,55].

**Figure 9:**
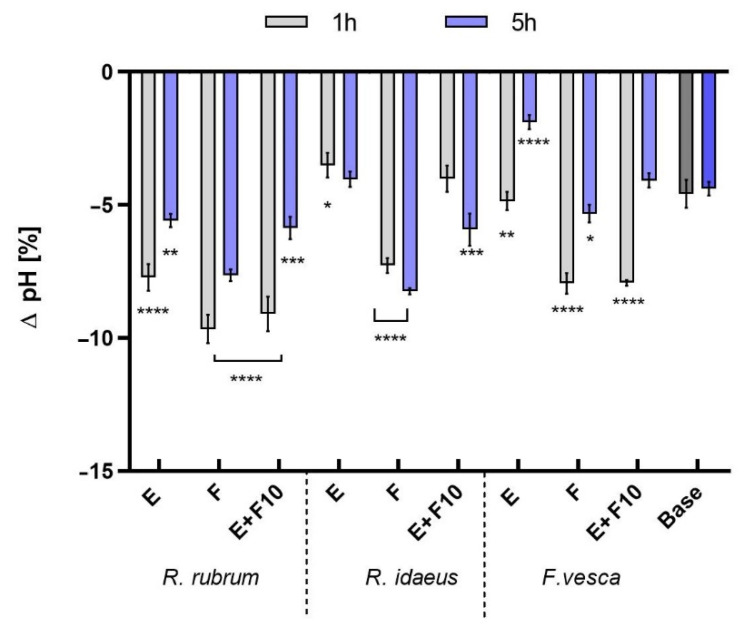
The influence of model skin tonics with *R. rubrum, R. idaeus,* and *F. vesca* extracts (E), ferments (F10), and extracts + ferments (E + F10) on skin pH. Data are the mean ± SD of three independent measurements. **** *p* < 0.0001, *** *p* < 0.001, ** *p* < 0.01 and * *p* < 0.05 compared to the base tonic.

In the next stage of the research, the influence of the analyzed samples on the skin pH was assessed. The tests were performed with the use of a skin pH meter. The correct pH of the skin is extremely important for keeping it in good condition. The physiological role of the acidic skin surface has historically been considered to be a defense mechanism against invading organisms. It has also been shown that several key enzymes that are involved in the synthesis and the maintenance of the proper skin barrier are highly dependent on the pH [56,57,58]. The pH analysis showed noticeable differences between the model tonics with redberry extracts and/or ferments. The zero level indicates no change in the skin pH relative to the control field, that is, the physiological pH of the test volunteers. It was observed that all of the tested formulations lowered the skin pH value in relation to the control field (Figure 9). The lowest deviation from the physiological pH of the skin was observed for the tonics with the addition of the extracts, while the greatest differences were shown by the preparations with 1% ferment, due to the acidic environment of the ferment itself. The greatest reduction in the skin pH was noticed for the formulation containing the *R. rubrum* extract and the ferment, achieving differences of about −8 compared to the control field after one hour of the application. The results that were obtained indicate that the ferments are not only a rich source of active ingredients, such as antioxidants, but they also have a positive effect on the pH of the skin, lowering it. This property is particularly important for the use of facial and body cleansing cosmetics, as an increase in the skin pH is observed due to the surfactants that are contained in their formulations. The ferments, as well as a consortium of ferments and extracts that are added to skin toners, can help the skin to return to a physiological pH.

## 3. Materials and Methods

### 3.1. Plant Material and Fermentation Procedure

Fruits of *R. rubrum, R. idaeus,* and *F. vesca* were collected on controlled and organic plantations. No chemical fertilizers or plant protection products were used in the cultivation. In addition, a preliminary selection was carried out after harvesting the plant material, paying particular attention to chemotaxonomic factors. The kombucha tea fungus starter was purchased from a commercial source from Poland. Initially, the berry extracts were prepared in a sterile beaker by mixing 15 g of fresh fruit and 500 mL of purified water at room temperature. The extracts were obtained by ultrasound-assisted extraction (UAE). The UAE was performed according to the method that was described by Yang et al. [59] in an ultrasonic bath (Digital Ultrasonic Cleaner, Berlin, Germany) equipped with a time controller, extracting for 30 min. Then, for fermentation, 50 g of sucrose (final concentration 10.0% *m*/*v*) was added to the resulting extracts and was filtered through membrane filters into sterile glass beakers (1000 mL, height 18 cm, diameter 8 cm). Tea mushrooms (3 g) and kombucha (50 mL) were added to the filtrate and fermentation was carried out for 10 and 20 days (in separate beakers) at room temperature (approximately 25 °C). After completion of the fermentation, the resulting kombucha was filtered. Ferments obtained after 10 days were designated as F10 and after 20 days as F20.

### 3.2. Determination of Biologically Active Compounds

The main metabolites (phenolic acids and flavonoids) were identified by using an ultra-high performance liquid chromatography (UHPLC) Infnity Series II with a DAD detector and Agilent 6224 ESI/TOF mass detector (Agilent Technologies, Santa Clara, CA, USA). The HPLC conditions were as follows: an RP18 reversed-phase column Titan (Supelco, Sigma-Aldrich, Burlington, MA, USA) (10 cm, 2.1 mm i.d., 1.9 µm particle size), a thermostat temperature of 30 °C, and a flow rate of 0.2 mL/min. A mixture of water with 0.05% of formic acid (solvent A) and acetonitrile with 0.05% of formic acid (solvent B) was used as a mobile phase. The compounds were separated using gradient elution according to the following program: 0–9 min from 98% A to 95% A (from 2% to 5% B), 9–24 min from 95% A to 92% A (from 5%to 8% B), 24–45 min from 92% A to 85% A (from 8% to 15% B), and 45–60 min from 85% A to 70% A (from 15% B to 30% B). Chromatograms were recorded from 200 to 400 nm. For the LC–MS analysis, the ion source operating parameters were as follows: drying gas temperature 325 °C, drying gas flow 8 L/min, nebulizer pressure 30 psi, capillary voltage 3500 V, fragmentator 170 V, and skimmer 65 V. Ions were acquired in the range of 100 to 1050 *m*/*z*.

### 3.3. Determination of Antioxidant Properties

#### 3.3.1. DPPH Radical Scavenging Assay

The ability of the obtained extracts to scavenge free radicals was determined using the method described by Brand-Williams et al. [60]. This method is based on using the 1,1-diphenyl-2-picrylhydrazyl (DPPH) radical. A total of 100 µL of water solutions of analyzed extracts and kombucha ferments at concentrations of 30, 300, 750, 1500, and 3000 µg/mL were transferred to a 96-well plate. Then, 100 µL of ethanol solution of DPPH was added to the samples and mixed. The absorbance was measured at a wavelength of 517 nm, every 5 min for 20 min, using a UV-VIS Filter Max spectrophotometer (Thermo Fisher Scientific, Waltham, MA, USA). The measurements were carried out in triplicate for each extract sample. The antioxidant capacity was expressed as a percentage of DPPH inhibition using Equation (1). The IC_50_ parameter was then determined, which determines the concentration of the extract or ferment that causes a 50% decrease in the initial concentration of the radical.

(1) $$\%\mathrm{DPPH}\mathrm{scavenging}=\frac{\mathrm{Abs}\mathrm{control}-\mathrm{Abs}\mathrm{sample}}{\mathrm{Abs}\mathrm{control}}\times 100$$

where Abs sample—absorbance of the sample; Abs control—absorbance of the control sample.

#### 3.3.2. ABTS• + Scavenging Assay

In order to determine the antioxidant properties of the water fruit extracts, the ABTS• + Scavenging Assay, which was described by Nicolas et al. [61], was performed. In the first step, 7 mM aqueous ABTS solution and 2.4 mM potassium persulfate were made. The prepared solutions were mixed in equal proportions and left at room temperature in darkness for 16 h. After this time, the solution was diluted in methanol to the absorbance level of about 1.0 (λ = 734 nm). Then, 1 mL of the analyzed extracts and ferments in concentrations of 30, 300, 750, 1500, and 3000 µg/mL was mixed with 1 mL of ABTS. The absorbance was measured at the wavelength λ = 734 nm using an Aquamate Helton spectrophotometer. The ABTS•+ scavenging was calculated using Equation (2). The IC_50_ parameter was then determined, which determines the concentration of the extract or ferment that causes a 50% decrease in the initial concentration of the radical.

(2) $$\%\mathrm{of}\mathrm{ABTS}•+\;\mathrm{scavenging}=1-\frac{\mathrm{As}}{\mathrm{Ac}}\times 100$$

where As—absorbance of the sample; Ac—absorbance of the control sample.

#### 3.3.3. Detection of Intracellular Levels of Reactive Oxygen Species (ROS)

Another analysis determining the antioxidant properties was carried out with the use of fluorogenic dye H_2_DCFDA. This analysis allows us to determine the ability of tested substances in terms of intracellular production of reactive oxygen species in cells. H_2_DCFDA has the ability to penetrate inside the cell, where it is transformed into a non-fluorescent compound. If reactive oxygen species are present in the cell, this compound is then transformed into highly fluorescent DCF. To determine the intracellular level of ROS in HaCaT and BJ, the cells were set in 96-well plates and cultured in an incubator for 24 h. Next, the DMEM medium was removed and replaced with 10 µM H_2_DCFDA (Sigma Aldrich, St. Louis, MO, USA) dissolved in serum free DMEM medium. The cells were incubated for 45 min and then incubated with the extracts and ferments in the concentrations of 30 and 300 µg/mL. The cells that were treated with 1 mM hydrogen peroxide (H_2_O_2_) were used as positive controls. The control samples were the cells that were not with the tested extracts. The fluorescence of DCF was measured every for 90 min using a FilterMax F5 microplate reader (Thermo Fisher Scientific, Waltham, MA, USA) at a maximum excitation of 485 nm and emission spectra of 530 nm [62].

### 3.4. Cytotoxicity Anlysis

#### 3.4.1. Cell Culture

In order to determine the cytotoxic activity of the analyzed extracts and ferments, two types of skin cells were used: fibroblasts (American Type Culture Collection Manassas, VA, USA) and keratinocytes (CLS Cell Lines Service GmbH, Eppelheim, Germany). The cells were grown in Dulbecco’s Modification of Eagle’s Medium (DMEM, Biological Industries, Cromwell, CO, USA) with high glucose content (4.5 g/L). The medium was enriched with sodium pyruvate, L-glutamine, 10% fetal bovine serum (Gibco, Waltham, MA, USA), and 1% antibiotics (100 U/mL penicillin and 1000 µg/mL streptomycin, Gibco). The cells were grown in an incubator in a humidified atmosphere of 95% air and 5% carbon dioxide and at 37 °C. After obtaining the required confluence, the medium was removed. The cells were washed with sterile phosphate buffered saline and then were detached from the bottom of the culture flasks with trypsin. Next, the cells were placed in fresh medium, plated in 96-well plates, and incubated for 24 h. After this time, the cells were treated with analyzed extracts and kombucha ferments in concentrations of 30 and 300 µg/mL and incubated for another 24 h. The effect of the model skin tonics on the viability and metabolism of keratinocytes at dilutions of 0.1 and 1% were also investigated. The tested formulations containing the extracts and ferments were incubated for 4 h. The control samples were the cells that were not treated with extracts and ferments.

#### 3.4.2. Alamar Blue Assay

The first test used to evaluate the viability of skin cells was Alamar Blue Assay. After the incubation of the cells that were treated with the extracts and ferments, the analyzed samples were removed from the wells and then resazurin solution (60 µM) was added. Plates were placed in an incubator for 2 h at 37 °C. Then, fluorescence was measured at wavelength λ = 570 nm using a FilterMax F5 microplate reader (Thermo Fisher Scientific, Waltham, MA, USA).

#### 3.4.3. Neutral Red Uptake Assay

The second test used to evaluate the viability of skin cells was Neutral Red Uptake. After incubation, the analyzed samples were removed from the wells and then the Neutral Red dye (40 µg/mL) was added to the wells. Plates were placed in an incubator for 2 h at 37 °C, then the Neutral Red dye was removed, and the cells were washed with phosphate buffered saline. After this, the phosphate buffered saline was removed and 150 µL of decolorizing buffer was added. The absorbance measurements were performed at wavelength λ = 540 nm using a FilterMax F5 microplate reader (Thermo Fisher Scientific, Waltham, MA, USA).

### 3.5. Transepidermal Water Loss (TEWL), Skin Hydration, and Skin pH Measurements

The TEWL, skin pH, and skin hydration measurements were conducted using TEWAmeter TM 300 probe, skin pHmeter PH950, and Corneometer CM825 probe connected to a MPA adapter (Courage + Khazaka Electronic, Köln, Germany), respectively. This study was conducted on 10 volunteers, according to the procedure described by Nizioł-Łukaszewska et al. [63]. The areas (2 × 2 cm in size) were marked on the forearm skin of volunteers. Then, 0.2 mL of the cosmetic was applied to 3 fields. One field (control field) was not treated with any sample. The sample solutions were gently spread over every skin fragment in the marked area and, after 20 min, dried with a paper towel. After 1 h and 5 h, the hydration, TEWL, and skin pH measurements were taken. The final result was the arithmetic mean (from each volunteer) of 5 independent measurements (skin hydration and pH) and 20 measurements (TEWL).

### 3.6. Preparation of the Model Skin Tonics

The final composition of the analyzed model moisturizing skin tonics is shown in Table 5. The raw materials that are widely used in the cosmetics industry were used to prepare the samples. A 500 g tonic was prepared according to the following procedure: purified water was poured into a glass beaker, and gluconolactone and sodium benzoate were dissolved and stirred with a mechanical stirrer (Chemland O20) until completely dissolved. The resulting basic tonic was divided into 10 equal parts. One was a model of the base tonic with no added extract and ferments. *R. rubrum*, *R. idaeus*, and *F. vesca* extracts/ferments were added to the remaining samples at a concentration of 1 wt.% in each sample.

### 3.7. Statistical Analysis

The data are presented as means ± SD of three independent experiments, in which each tested concentration of individual samples was repeated three times, hence the number “n” from all experiments was 9. The obtained experimental data were analyzed with one-way analysis of variance (ANOVA) followed by Tukey’s multiple comparison test. The statistical significance was determined at **** *p* < 0.0001, *** *p* < 0.001, ** *p* < 0.01, and * *p* < 0.05 compared to the control. The statistical analysis was performed using GraphPad Prism 8.0.1 (GraphPad Software, Inc., San Diego, CA, USA).

## 4. Conclusions

Berry plant fruit extracts are known for their pro-health properties and have applications in the cosmetic industry as active ingredients. This study has shown that, in addition to the fruit extracts of *R. rubrum, F. vesca,* and *R. idaeus*, fermented plant extracts also offer a wide range of possibilities. There is a growing amount of literature indicating that substances other than black or green tea can be a good substrate for kombucha fermentation. This study has shown that fruit extracts that are fermented with kombucha have a higher content of biologically active compounds than aqueous extracts alone. These values correlate with their antioxidant potential. The fermentation time was shown to affect the content and the properties of the samples. The ferments after 20 days of fermentation have a higher ability to inhibit the DPPH radical and the ABTS. In turn, this study of cytotoxicity against skin cells: fibroblasts and keratinocytes, showed a more favorable effect of F10 on the cell viability and proliferation. By prolonging the fermentation time, the ferments showed a tendency to inhibit skin cell proliferation. The work also included making model skin tonics containing the tested extract and the ferment, as well as a combination of the extract and the ferment in a 1:1 ratio. The formulations were shown to have a positive effect on skin hydration and pH, lowering the latter. The results that have been obtained indicate that ferments, in addition to probiotic activity supporting the beneficial microorganisms inhabiting human skin, can also be a valuable ingredient present in pharmaceutical and cosmetic products.

## Figures and Tables

**Table 1:** Bioactive Compounds Detected Using UHPLC/DAD/ESI-MS in *R. rubrum*, *R. ideaus,* and *F. vesca*.

Molecular Formula	Name of Compound	*R. idaeus*	*R. rubrum*	*F. vesca*
C_6_H_12_O_7_	Gluconic acid	x	x	x
C_7_H_12_O_6_	Quinic acid		x	x
C_10_H_16_O_16_	Citric acid derivative	x		
C_6_H_8_O_7_	Citric acid	x		
C_7_H_6_O_5_	Galic acid	x	x	x
C_14_H_16_O_10_	Galloyl quinic acid	x	x	x
C_30_H_26_O_14_	Prodelphinidin B4/B3		x	
C_13_H_16_O_8_	Hydroxybenzoic acid -hexoside	x	x	
C_15_H_14_O_7_	Gallocatechin		x	
C_14_H_18_O_9_	Methyldihydroxybenzo -icacid hexoside	x	x	
C_11_H_12_O_2_N_2_	Tryptophan			x
C_15_H_18_O_9_	Caffeoyl glucose		x	
C_15_H_18_O_8_	Coumaroyl hexoside		x	
C_15_H_18_O_9_	Caffeic acid hexosie	x		
C_15_H_14_O_7_	Epigallocatechin	x	x	
C_15_H_14_O_6_	Catechin	x	x	
C_15_H_18_O_9_	Caffeoyl glucose		x	
C_16_H_18_O_9_	Chlorogenic acid		x	x
C_27_H_30_ O_16_	Cyanidin 3-sophoroside	x	x	
C_33_H_40_O_20_	Cyanidin-3-glucosyl -rutinoside		x	
C_26_H_28_O_15_	Cyanidin 3-sambubioside		x	
C_21_H_20_O_11_	Cyanidin 3-glucoside	x		
C_15_H_14_O_6_	Epicatechin	x	x	x
C_32_H_38_O_19_	Cyanidin 3-xylosylrutinoside		x	
C_22_H_18_O_11_	Epigallocatechin gallate	x	x	x
C_21_H_20_O_10_	Pelargonidin-3-O -glucoside			x
C_21_H_22_O_11_	Ferulic acid hexose -derivative			x
C_7_H_6_O_3_	Salicylic acid		x	
C_21_H_20_O_13_	Unknown flavonoid		x	
C_22_H_18_O_10_	Epicatechingallate /catechingallate		x	
C_20_H_16_O_12_	Ellagic acid rhamnoside			x
C_14_H_6_O_8_	Ellagic acid			x
C_20_H_20_O_11_	Taxifolin 3-alpha-L-arabino -furanoside			x
C_27_H_30_O_16_	Rutoside	x	x	x
C_21_H_20_O_13_	Quercetin 3-O-glucuronide	x		x
C_21_H_20_O_12_	Quercetin glucoside		x	x
C_21_H_20_O_11_	Kaempferol hexoside		x	x
C_21_H_18_O_12_	Methylellagic acid -rhamnoside			x
C_21_H_18_O_12_	Methylellagic acid hexose			x

x indicates the occurrence of the detected compounds in the tested plant.

**Table 2:** UHPLC/DAD/ESI-MS quantitative analysis of *R. rubrum*, *R. ideaus,* and *F. vesca* water extracts and kombucha ferments. Values are means ± standard deviation (SD) of triplicate.

Analyzed Plant	Name of Compound	Content (µg/mL)		
		Extract F10 (10 Days) F20 (20 Days)		
*Rubus idaeus*	Gallic acid	-	3.17 ± 0.03	3.82 ± 0.04
	Caffeoyl glucose I	0.20 ± 0.00	0.22 ± 0.00	0.27 ± 0.01
	Salicylic acid glucoside	10.03 ± 0.04	10.33 ± 0.14	10.95 ± 0.06
	Benzoic acid hexoside	2.48 ± 0.10	2.78 ± 0.05	2.72 ± 0.13
	Rutoside	-	0.20 ± 0.00	0.36 ± 0.00
	Quercetin 3-O-glucuronide	0.10 ± 0.00	0.10 ± 0.00	0.12 ± 0.00
	Quercetin glucoside	-	0.23 ± 0.00	0.17 ± 0.00
	Kaempferol rutoside	-	0.27 ± 0.01	0.26 ± 0.01
	Galloyloquinic	-	0.87 ± 0.02	1.12 ± 0.04
	Gallocatechin (I)	-	2.49 ± 0.04	2.65 ± 0.08
	Gallocatechin (II)	-	4.42 ± 0.18	5.60 ± 0.14
	Catechin	-	1.83 ± 0.02	2.02 ± 0.05
	Epicatechin	-	0.55 ± 0.02	0.60 ± 0.01
	Epigallocatechingallate	-	3.90 ± 0.14	4.96 ± 0.18
*Ribes rubrum*	Gallic acid	-	2.48 ± 0.08	2.84 ± 0.10
	Chlorogenic acid	-	0.15 ± 0.01	0.31 ± 0.01
	Benzoicacid hexoside	3.92 ± 0.20	3.78 ± 0.12	3.69 ± 0.14
	Methyldihydroxybenzoicacidhexoside	0.36 ± 0.01	0.28 ± 0.01	0.30 ± 0.01
	Caffeoylyglucose	0.14 ± 0.01	0.15 ± 0.00	0.14 ± 0.00
	Rutoside	-	0.89 ± 0.01	1.01 ± 0.02
	Quercetin hexoside	-	0.20 ± 0.01	0.15 ± 0.00
	Kaempferol hexoside	-	0.24 ± 0.01	0.20 ± 0.01
	Galloyloquinic	-	0.96 ± 0.03	0.95 ± 0.04
	Gallocatechin (I)	0.15 ± 0.01	0.63 ± 0.03	0.87 ± 0.02
	Gallocatechin (II)	-	0.44 ± 0.02	0.82 ± 0.02
	Catechin	-	1.19 ± 0.01	1.10 ± 0.00
	Epicatechin	-	1.97 ± 0.04	1.49 ± 0.01
	Epigallocatechin gallate	-	0.39 ± 0.01	0.74 ± 0.02
*Fragaria vesca*	Gallic acid	0.32 ± 0.02	2.94 ± 0.11	3.85 ± 0.01
	Chlorogenic acid	-	0.12 ± 0.00	0.23 ± 0.01
	Rutoside	-	0.61 ± 0.01	0.78 ± 0.01
	Taxifolin	0.29 ± 0.00	0.87 ± 0.04	0.89 ± 0.03
	Quercetin glucuronide	0.10 ± 0.00	-	-
	Quercetin hexoside	0.04 ± 0.00	0.25 ± 0.01	0.27 ± 0.00
	Kaempferol hexoside	-	0.09 ± 0.00	0.13 ± 0.00
	Ellagic acid rhamnoside	0.15 ± 0.00	-	-
	Ellagic acid	-	0.15 ± 0.00	0.17 ± 0.00
	Methyl ellagic acid rhamnoside	1.96 ± 0.08	0.67 ± 0.03	0.75 ± 0.02
	Methyl ellagic acid hexose	0.24 ± 0.01	-	-
	Galloyloquinic	-	0.85 ± 0.01	1.03 ± 0.05
	Gallocatechin (I)	-	0.91 ± 0.04	0.75 ± 0.03
	Gallocatechin (II)	-	0.98 ± 0.04	0.91 ± 0.00
	Catechin	-	0.28 ± 0.01	0.28 ± 0.01
	Epicatechin	-	0.80 ± 0.03	0.73 ± 0.03
	Epigallocatechin gallate	-	1.09 ± 0.04	1.03 ± 0.05

**Table 3:** Values of IC_50_ of DPPH radical scavenging for *R. rubrum*, *R. ideaus*, and *F. vesca* extracts and kombucha ferments after 20 min of exposure. Values are means ± standard deviation (SD) of triplicate.

	Plant Extract	Ferment 10 Days	Ferment 20 Days
Type of Plant Analyzed	IC_50_ [µg/mL]		
*R. rubrum*	3207.45 ± 34.48	1661.29 ± 15.28 ****	1730.95 ± 19.56 ****
*R. idaeus*	3246.56 ± 31.39	1871.61 ± 18.45 ****	1789.21 ± 17.45 ****
*F. vesca*	2165.26 ± 23.46	1740.81 ± 16.71 ****	1846.82 ± 17.47 ****

where: **** *p* < 0.0001 compared to the Plant Extract.

**Table 4:** Values of IC_50_ of ABTS+ radical scavenging for *R. rubrum*, *R. ideaus*, and *F. vesca* extracts and kombucha ferments after 20 min of exposure. Values are means ± standard deviation (SD) of triplicate.

	Plant Extract	Ferment 10 Days	Ferment 20 Days
Type of Plant Analyzed	IC_50_ [µg/mL]		
*R. rubrum*	270.81 ± 2.23	129.18 ± 1.58 ****	124.71 ± 1.25 ****
*R. idaeus*	124.32 ± 1.41	130.49 ± 1.26 **	120.84 ± 1.35 ****
*F. vesca*	125.48 ± 1.27	132.41 ± 2.43 **	127.20 ± 1.14 ****

where: **** *p* < 0.0001, ** *p* < 0.01 compared to the Plant Extract.

**Table 5:** Formulation of the analyzed model skin tonics.

INCI Name	Concentration [wt.%]
Aqua	88.5
Propanediol	2.0
Sodium Hyaluronate	1.5
Sorbitol	1.0
Niacinamide	2.0
D-Panthenol	1.5
Extract/Ferment/Extract + Ferment	1.0
Lactobionic Acid	1.0
Gluconolactone and Sodium Benzoate	1.5

## Data Availability

Data are contained within the article.

## Supplementary Materials

The following are available online at: https://www.mdpi.com/article/10.3390/ijms232314675/s1, Table S1. Characterization of metabolites identified in *Ribes idaeus* extract and ferment by HPLC-MS in negative and positive ion mode; Table S2. Characterization of metabolites identified in *Ribes rubrum* extract and ferment by HPLC-MS in negative and positive ion mode; Table S3. Characterization of metabolites identified in *Fragaria vesca* extract and ferment by HPLC-MS in negative and positive ion mode.
