# Supplementary material for: Assessment of Cosmetic and Dermatological Properties and Safety of Use of Model Skin Tonics with Kombucha-Fermented Red Berry Extracts
Source: Int J Mol Sci. 2022 Nov 24;23(23):14675. doi: 10.3390/ijms232314675 (PMC9741178; doi:10.3390/ijms232314675)
Supplement: Supplementary file 1 [file ijms-23-14675-s001.zip › ijms-2019489-supplementary.pdf]

International Journal of *Molecular Sciences*

MDPI

Supplementary material

Assessment of Cosmetic and Dermatological Properties and Safety of Use of Model Skin Tonics with Kombucha-Fermented Red Berry Extracts

Aleksandra Ziemlewska [1], Zofia Nizioł-Łukaszewska [1], Martyna Zagórska-Dziok [1], Magdalena Wójciak [2], Dariusz Szczepanek [3] and Ireneusz Sowa [2,*]

[1] Department of Technology of Cosmetic and Pharmaceutical Products, Medical College, University of Information Technology and Management in Rzeszow, Sucharskiego 2, 35-225 Rzeszow, Poland

[2] Department of Analytical Chemistry, Medical University of Lublin, Aleje Raclawickie 1, 20-059 Lublin, Poland

[3] Chair and Department of Neurosurgery and Paediatric Neurosurgery, Medical University of Lublin, 20-090 Lublin, Poland

* Correspondence: i.sowa@umlub.pl

Table S1. Characterization of metabolites identified in *Ribes idaeus* extract (E) and ferment (F) by UHPLC-MS in negative ion mode

Rt (min.)	m/z-H	Error (ppm)	Fragments	Formula	Compound	Identification
1.58	195.05121	0.89	-	$C_6H_{12}O_7$	gluconic acid (F)	std
2.28	391.03821	4.21	191	$C_{10}H_{16}O_{16}$	citric acid derivative (E)	[1]
2.52	191.02011	2.0	-	$C_6H_8O_7$	citric acid (E)	[1],(std)
4.86	169.01455	1.78	-	$C_7H_6O_5$	galic acid (F)	std
6.16	343.06870	4.74	191	$C_{14}H_{16}O_{10}$	galloylquinic acid (F)	[2]*****
6.42	299.07822	3.26	137	$C_{13}H_{16}O_8$	hydroxybenzoic acid hexoside (F,E)	[1]*
9.17	299.07871	4.60	137	$C_{13}H_{16}O_8$	hydroxybenzoic acid hexoside (E)	[1]*
9.44	329.08925	4.38	167	$C_{14}H_{18}O_9$	methyl dihydroxybenzoic acid hexoside (E)	[3]**
9.44	305.06811	4.68	125,169	$C_{15}H_{14}O_7$	epigallocatechin (F)	[4]******
12.47	341.08901	3.52;	161,179	$C_{15}H_{18}O_9$	caffeic acid hexoside (F,E)	[4]***
14.61	305.06818	4.91	125,169	$C_{15}H_{14}O_7$	epigallocatechin (F)	[2]******
15.56	289.07202	0.89	221,245	$C_{15}H_{14}O_6$	catechin (F)	[4], (std)
17.83	609.14781	2.79	-	$C_{27}H_{30}O_{16}$	cyanidin 3-sophoroside (F,E)	[5]
18.75	447.09322	2.32	-	$C_{21}H_{20}O_{11}$	cyanidin 3-glucoside (F,E)	[5]
19.39	289.07211	1.20	221,245	$C_{15}H_{14}O_6$	epicatechin (F)	[4], (std)
20.64	457.07926	3.53	125,169,305	$C_{22}H_{18}O_{11}$	epigallocatechin gallate (F)	[2]******
32.32	609.14912	4.94	300, 463	$C_{27}H_{30}O_{16}$	rutin (F)	[4], (std)
32.82	477.06911	3.44	301	$C_{21}H_{18}O_{13}$	quercetin 3-O-glucuronide (F,E)	[4]****

*Quantification was based on calibration curve for p-hydroxybenzoic acid, **Quantification was based on calibration curve for dihydroxybenzoic acid, ***Quantification was based on calibration curve for caffeic acid, ****Quantification was based on calibration curve for quercetin glucoside, *****Quantification was based on calibration curve for gallic acid, ******Quantification was based on calibration curve for catechin, std - standard

Table S2. Characterization of metabolites identified in *Ribes rubrum* extract and ferment by UHPLC-MS in negative ion mode

Rt (min.)	m/z-H	Error (ppm)	Fragments	Formula	Compound	Identifi-cation
1.57	195.05130	1.40	-	$C_6H_{12}O_7$	gluconic acid (F)	std
1.60	191.05624	0.67	-	$C_7H_{12}O_6$	quinic acid (E)	[6], (std)
4.60	169.01469	2.61	-	$C_7H_6O_5$	galic acid (F)	std
5.83	343.06751	1.25	191	$C_{14}H_{16}O_{10}$	galloylquinic acid (F)	[2]*****
5.93	609.12601	1.69	-	$C_{30}H_{26}O_{14}$	prodelphinidin B4/B3 (E)	[6]
8.72	299.07789	2.16	137	$C_{13}H_{16}O_8$	hydroxybenzoic acid hexoside (F,E)	[3]*
9.07	305.06697	0.96	125	$C_{15}H_{14}O_7$	gallocatechin (F,E)	[3]**
10.80	329.08917	4.13	167	$C_{14}H_{18}O_9$	methyl dihydroxybenzoic acid hexoside (F,E)	[3]****
12.39	341.08811	0.89	161, 179;	$C_{15}H_{18}O_9$	caffeoylglucose (F,E)	[3,6]***
12.56;	325.09423	4.11	163	$C_{15}H_{18}O_8$	coumaroyl hexoside (E)	[3,7]******
12.99	341.08798	0.51	179, 135	$C_{15}H_{18}O_9$	caffeoylglucose (F,E)	[3,6]***
14.16	305.06804	4.46	169, 125	$C_{15}H_{14}O_7$	epigallocatechin (F)	[2]**
14.76	325.09331	1.28	163	$C_{15}H_{18}O_8$	coumaroyl hexoside (E)	[3,7]******
15.01	289.07201	0.86	221,245	$C_{15}H_{14}O_6$	catechin (F)	std
15.21	341.08824	1.27	135, 179	$C_{15}H_{18}O_9$	caffeoylglucose (E)	[6]***
15.80	353.08901	3.4	191,179	$C_{16}H_{18}O_9$	chlorogenic acid (F)	std
18.09	609.14691	1.31	-	$C_{27}H_{30}O_{16}$	cyanidin 3-sophoroside (F,E)	[8]
18.79	755.20311	0.25	-	$C_{33}H_{40}O_{20}$	cyanidin-3-glucosylrutinoside (F,E)	[8]
18.91	579.13567	0.22	-	$C_{26}H_{28}O_{15}$	cyanidin 3-sambubioside (F,E)	[8]
18.96	289.07297	4.17	221,245	$C_{15}H_{14}O_6$	epicatechin (F)	std
19.91	725.19524	2.46	-	$C_{32}H_{38}O_{19}$	cyanidin 3-xylosylrutinoside (F,E)	[8]
20.14	457.07989	4.92	125,169,305	$C_{22}H_{18}O_{11}$	epigallocatechin gallate (F)	[2]**
25.52	137.02606	4.65	-	$C_7H_6O_3$	salicylic acid (E)	[3], (std)
26.34	479.083698	1.22	316	$C_{21}H_{20}O_{13}$	unknown flavonoid (F)	-
28.42	441.083611	2.01	289,169,125	$C_{22}H_{18}O_{10}$	epicatechin gallate/catechin gallate (F)	[9]**
31.79	609.14679	1.12	300	$C_{27}H_{30}O_{16}$	rutin (F,E)	[3], (std)
32.92	463.08997	3.81	300	$C_{21}H_{20}O_{12}$	quercetin glucoside (F)	std
37.72	447.09461	2.96	284	$C_{21}H_{20}O_{11}$	kaempferol hexoside(F)	[6]

*Quantification was based on calibration curve for p-hydroxybenzoic acid, **Quantification was based on calibration curve for catechin, ***Quantification was based on calibration curve for caffeic acid, ****Quantification was based on calibration curve for dihydroxybenzoic acid, *****Quantification was based on calibration curve for gallic acid, ******Quantification was based on calibration curve for p-coumaric acid, std – standard

Table S3. Characterization of metabolites identified in *Fragaria vesca* extract and ferment by HPLC-MS in negative ion mode

Rt (min.)	m/z-H	Error (ppm)	Frag-ments	Formula	Compound	Identification
1.57	195.05130	1.40	-	$C_6H_{12}O_7$	gluconic acid (F)	std
1.60	191.05664	2.75	-	$C_7H_{12}O_6$	quinic acid (E)	std
4.78	169.01462	2.19	125	$C_7H_6O_5$	gallic acid (F,E)	std
6.10	343.06801	2.73	191,169	$C_{14}H_{16}O_{10}$	galloylquinic acid (F)	[2]***
9.42	305.06805	4.49	125	$C_{15}H_{14}O_7$	epigallocatechin (F)	[2]****
10.14	203.0831	2.44	142	$C_{11}H_{12}O_2N_2$	Tryptophan (E)	[10]
14.60	305.06784	3.80	169	$C_{15}H_{14}O_7$	epigallocatechin (F)	[2]****
15.48	289.07278	3.51	221,245	$C_{15}H_{14}O_6$	catechin (F)	std
16.23	353.08911	3.68	191,179	$C_{16}H_{18}O_9$	chlorogenic acid (F)	std
19.38	289.073021	4.34	221,245	$C_{15}H_{14}O_6$	epicatechin (F)	std
20.29	431.09715	0.21	268	$C_{21}H_{20}O_{10}$	pelargonidin-3-O-glucoside (F,E)	[11]
20.59	457.07973	4.57	125,169,305	$C_{22}H_{18}O_{11}$	epigallocatechin gallate (F)	[2]****
22.55	449.11001	2.39	289,267	$C_{21}H_{22}O_{11}$	ferulic acid hexose derivative (F,E)	[10]
30.86	447.05881	4.26	300	$C_{20}H_{16}O_{12}$	ellagic acid rhamnoside (F,E)	[11,12]*
31.87	300.99851	-1.59	-	$C_{14}H_6O_8$	ellagic acid (F,E)	[11]
32.18	435.09526	4.53	285	$C_{20}H_{20}O_{11}$	taxifolin 3-alpha-L-arabinofuranoside (F,E)	[10,11]**
32.32	609.14629	0.3	300, 463	$C_{27}H_{30}O_{16}$	rutin (F)	std
32.93	477.06900	3.21	300	$C_{21}H_{18}O_{13}$	quercetin glucuronide (F,E)	[10]**
33.56	463.09002	3.92	300	$C_{21}H_{20}O_{12}$	quercetin glucoside (F,E)	[10], (std)
39.13	447.09482	3.43	284	$C_{21}H_{20}O_{11}$	kaempferol hexoside (F,E)	[10]
40.56	461.07100	-3.35	300, 315	$C_{21}H_{18}O_{12}$	methylellagic acid rhamnoside (F,E)	[10]*
42.25	461.07112	-3.09	300, 315	$C_{21}H_{18}O_{12}$	methylellagic acid hexose (F,E)	[10]*

*Quantification was based on calibration curve for ellagic acid, **Quantification was based on calibration curve for quercetin glucoside, ***Quantification was based on calibration curve for gallic acid, ****Quantification was based on calibration curve for catechin; str - standard

References

1. Paudel, L.; Wyzgoski, F.J.; Scheerens, J.C.; Chanon, A.M.; Reese, R.N.; Smiljanic, D.; Wesdemiotis, C.; Blakeslee, J.J.; Riedl, K.M.; Rinaldi, P.L. Nonanthocyanin Secondary Metabolites of Black Raspberry (Rubus Occidentalis L.) Fruits: Identification by HPLC-DAD, NMR, HPLC-ESI-MS, and ESI-MS/MS Analyses. *J. Agric. Food Chem.* **2013**, doi:dx.doi.org/10.1021/jf4039953.
2. Romani, A.; Campo, M.; Pinelli, P. HPLC/DAD/ESI-MS Analyses and Anti-Radical Activity of Hydrolyzable Tannins from Different Vegetal Species. *Food Chem.* **2012**, *130*, 214–221, doi:10.1016/j.foodchem.2011.07.009.
3. Zhao, Y.; Lu, H.; Wang, Q.; Liu, H.; Shen, H.; Xu, W.; Ge, J.; He, D. Rapid Qualitative Profiling and Quantitative Analysis of Phenolics in Ribes Meyeri Leaves and Their Antioxidant and Antidiabetic Activities by HPLC-QTOF-MS/MS and UHPLC-MS/MS. *J. Sep. Sci.* **2021**, *44*, 1404–1420, doi:10.1002/jssc.202000962.

4. Kashchenko, N.I.; Olennikov, D.N.; Chirikova, N.K. Metabolites of Siberian Raspberries: Lc-Ms Profile, Seasonal Variation, Antioxidant Activity, and Thermal Stability of Rubus Matsumuranus Phenolome. *Plants* **2021**, *10*, doi:10.3390/plants10112317.

5. Tumbas Šaponjac, V.; Gironés-Vilaplana, A.; Djilas, S.; Mena, P.; Ćetković, G.; Moreno, D.A.; Čanadanović-Brunet, J.; Vulić, J.; Stajčić, S.; Krunić, M. Anthocyanin Profiles and Biological Properties of Caneberry (Rubus Spp.) Press Residues. *J. Sci. Food Agric.* **2014**, *94*, 2393–2400, doi:10.1002/jsfa.6564.

6. D'Urso, G.; Montoro, P.; Piacente, S. Detection and Comparison of Phenolic Compounds in Different Extracts of Black Currant Leaves by Liquid Chromatography Coupled with High-Resolution ESI-LTQ-Orbitrap MS and High-Sensitivity ESI-Qtrap MS. *J. Pharm. Biomed. Anal.* **2020**, *179*, doi:10.1016/j.jpba.2019.112926.

7. Anttonen, M.J.; Karjalainen, R.O. High-Performance Liquid Chromatography Analysis of Black Currant (Ribes Nigrum L.) Fruit Phenolics Grown Either Conventionally or Organically. *J. Agric. Food Chem.* **2006**, *54*, 7530–7538, doi:10.1021/jf0615350.

8. Xianli Wu, Liwei Gu, Ronald L. Prior, S.M. Characterization of Anthocyanins and Proanthocyanidins in Some Cultivars of Ribes , Aronia , and Sambucus and Their Antioxidant Capacity. *J. Agric. Food Chem.* **2004**, *52*, 7846–7856.

9. Singh, A.; Bajpai, V.; Kumar, S.; Sharma, K.R.; Kumar, B. Profiling of Gallic and Ellagic Acid Derivatives in Different Plant Parts of Terminalia Arjuna by HPLC-ESI-QTOF-MS/MS. *Nat. Prod. Commun.* **2016**, *11*, 239–244, doi:10.1177/1934578x1601100227.

10. Sun, J.; Liu, X.; Yang, T.; Slovin, J.; Chen, P. Profiling Polyphenols of Two Diploid Strawberry (Fragaria Vesca) Inbred Lines Using UHPLC-HRMSn. *Food Chem.* **2014**, *146*, 289–298, doi:10.1016/j.foodchem.2013.08.089.

11. D'Urso, G.; Maldini, M.; Pintore, G.; D'Aquino, L.; Montoro, P.; Pizza, C. Characterisation of Fragaria Vesca Fruit from Italy Following a Metabolomics Approach through Integrated Mass Spectrometry Techniques. *Lwt* **2016**, *74*, 387–395, doi:10.1016/j.lwt.2016.07.061.

12. Del Bubba, M.; Checchini, L.; Chiuminatto, U.; Doumett, S.; Fibbi, D.; Giordani, E. Liquid Chromatographic/Electrospray Ionization Tandem Mass Spectrometric Study of Polyphenolic Composition of Four Cultivars of Fragaria Vesca L. Berries and Their Comparative Evaluation. *J. Mass Spectrom.* **2012**, *47*, 1207–1220, doi:10.1002/jms.3030.
